# Whole-genome DNA methylation profiling reveals epigenetic signatures in developing muscle in Tan and Hu sheep and their offspring

**DOI:** 10.3389/fvets.2023.1186040

**Published:** 2023-06-14

**Authors:** Caijuan Yue, Jiakang Wang, Yifei Shen, Junli Zhang, Jian Liu, Aiping Xiao, Yisha Liu, Hehua Eer, Qiao-e Zhang

**Affiliations:** ^1^College of Animal Science and Technology, Ningxia University, Yinchuan, Ningxia, China; ^2^Ningxia Academy of Agriculture and Forestry Sciences, Yinchuan, Ningxia, China; ^3^College of Life Science and Technology, Huazhong University of Science and Technology, Wuhan, Hubei, China; ^4^Institute of Marxism, China University of Geosciences, Wuhan, Hubei, China; ^5^Animal Husbandry Extension Station, Yinchuan, Ningxia, China

**Keywords:** sheep, muscle development, DNA methylation, whole-genome bisulfite sequencing, epigenetics

## Abstract

**Introduction:**

The Tan sheep is a popular local breed in China because of its tenderness and flavor. The Hu sheep breed is also famous for its high litter size, and its muscle growth rate is faster than that of Tan sheep. However, the epigenetic mechanism behind these muscle-related phenotypes is unknown.

**Methods:**

In this study, the longissimus dorsi tissue from 18 6 month-old Tan sheep, Hu sheep, and Tan-Hu F2 generation (6 sheep per population) were collected. After genomic DNA extraction, whole-genome bisulfite sequencing (WGBS) and bioinformatics analysis were performed to construct genome-wide DNA methylome maps for the Tan sheep, Hu sheep and their Tan-Hu F2 generation.

**Results:**

Distinct genome-wide DNA methylation patterns were observed between Tan sheep and Hu sheep. Moreover, DNA methylated regions were significantly increased in the skeletal muscle from Tan sheep vs. the F2 generation compared to the Hu sheep vs. F2 generation and the Tan sheep vs. Hu sheep. Compared with Hu sheep, the methylation levels of actin alpha 1 (*ACTA1*), myosin heavy chain 11 (*MYH11*), Wiskott-Aldrich syndrome protein (*WAS*), vav guanine nucleotide exchange factor 1 (*VAV*1), fibronectin 1 (*FN1*) and Rho-associated protein kinase 2 (*ROCK2*) genes were markedly distinct in the Tan sheep. Furthermore, Gene Ontology analysis indicated that these genes were involved in myotube differentiation, myotube cell development, smooth muscle cell differentiation and striated muscle cell differentiation.

**Conclusion:**

The findings from this study, in addition to data from previous research, demonstrated that the *ACTA1*, *MYH11*, *WAS*, *VAV1*, *FN1*, and *ROCK2* genes may exert regulatory effects on muscle development.

## 1. Introduction

High-quality mutton has become increasingly popular in recent years as living standards and dietary habits have greatly improved (1, 2). Tan sheep (*Ovis aries*) have become one of the most important sheep breeds used for both meat and wool production in China (3). It is recognized for its tender meat, unique flavor and lamb wool. Yanchi Tan sheep meat has been selected as State banquet designated ingredients for four times (2016 Hangzhou G20 Summit, 2017 Xiamen BRICS Summit, 2018 Qingdao SCO Summit and 2019 Summer Davos Annual Meeting in Dalian). Tan sheep is a short-tailed indigenous ovine breed from the Ningxia Hui Autonomous Region, China (4) and has become more widely distributed throughout China. However, due to the slow growth and low lambing rate of Tan sheep, the meat production performance of Tan sheep is seriously affected, which is far from meeting the huge demand of consumers for Tan sheep meat. In order to improve the productivity of Tan sheep meat, the Tan sheep Breeding Project Team introduced excellent Hu sheep for crossbreeding, in order to improve the production performance of Tan sheep without affecting the quality and flavor of Tan sheep. The Hu sheep is extensively reared for its high litter size (5, 6), and its muscle growth rate is faster than that of Tan sheep (7–9). Their hybrid offspring also grew faster than Tan sheep (data not published). However, the epigenetic mechanism that may be driving these muscle-related phenotypes (the faster growth for Hu sheep and more tender meat for Tan sheep) is unknown.

The most common form of DNA methylation is the addition of a methyl group to the fifth carbon of a cytosine base (5-mC), followed by adenine methylation (10, 11). Many studies have suggested that DNA methylation plays crucial roles in the maintenance of chromatin structure, DNA conformation and DNA stability as well as the regulation of cell growth and proliferation by controlling the expression of target genes (12–14). DNA methylation also exerts regulatory functions in X chromosome inactivation (15), gene imprinting (16), embryonic development (17), and human diseases (18). Previous studies have demonstrated that the quality and flavor of livestock and poultry meat is regulated by DNA methylation, gene expression, and other factors encoded by multiple genes (19–22). More recently, whole-genome DNA methylation analysis has been employed to assess phenotypic variations (e.g., growth rate, body size, metabolic rate, fat composition, and muscle development) in both humans and animals (23, 24). Several studies have shown that DNA methylation plays a key role in regulating skeletal muscle development of pig (25) and sheep (26, 27). In Hu sheep, the DMGS (differential methylation genes) may be involved in muscle growth and metabolism (26). Cao et al. analyzed genome-wide DNA methylation level in longissimus dorsi muscle between Small Tailed Han and Dorper × Small Tailed Han crossbred sheep. Their result shown that the mean carcass weight of the crossbred sheep (30.53 ± 2.60) was significantly higher than that of the Small Tailed Han sheep (24.33 ± 1.86), and the net meat weight, dressing percentage and meat percentage were also higher in the crossbred sheep than in the Small Tailed Han sheep (28).

Therefore, the purpose of this study was to reveal the potential epigenetic factors affecting the growth rate and meat quality of mutton using Tan sheep, Hu sheep and Tan-Hu hybrid F2 offspring by genomic DNA methylation sequencing. This study will serve as a valuable resource for DNA methylation investigations on screening candidate genes which might be related to meat production in Tan sheep.

## 2. Materials and methods

### 2.1. Ethics statement

All methods involving animal experiments were performed strictly in accordance with the recommendations for the Care and Use of Laboratory Animals of the National Institutes of Health of China. The protocol employed in this study was reviewed and approved by the Animal Ethical and Welfare Committee of the Animal Science Institute, Ningxia Academy of Agriculture and Forestry Sciences (approval number: AEWC-GAU-2019-001).

### 2.2. Animals and sample collection

Tan sheep are mainly distributed in Ningxia, Gansu, Inner Mongolia, Shaanxi, and areas adjacent to Ningxia in China. Six 3 month-old Tan ewes were selected from the core group of the Ningxia Yanchi Tan sheep breed conservation farm. Meanwhile, six 3 month-old Hu ewes and six 3 month-old Tan-Hu F2 generation were selected from a farm in Ningxia, then the 18 ewes were placed on the same diet until they were 6 months old. All ewes used in this study lived under the same conditions. When they were 6 months old, the ewes were humanely sacrificed using electro-stunning followed by severing of blood vessels in the neck. Longissimus dorsi tissues from the same position of each ewe were excised immediately from each carcass and then stored in liquid nitrogen until required.

### 2.3. DNA extraction and preparation for bisulfite sequencing

Genomic DNA extraction was performed using the TIANamp Genomic DNA Kit (Tiangen, Beijing, China) according to the manufacturer’s protocol. *RNase* treatment was carried out to remove RNA contaminants and ensure DNA purity. The purity and quantity of total DNA were assessed using the Bioanalyzer 2,100 system (Agilent, CA, United States). DNA samples with an *A*_260_/*A*_280_ ratio of 1.8–2.0 were considered of acceptable quality and were used for further analysis. The DNA of 6 sheep from each group was combined into a pool for library construction.

The DNA libraries for whole-genome bisulfite sequencing (WGBS) were prepared as described previously (24, 29). Briefly, genomic DNA was fragmented into small segments (100–300 bp) using a sonicator (Covaris Inc., MA, United States), and then purified using the MiniElute PCR Purification Kit (QIAGEN, Hilden, Germany). After end-repair and A-tailing, the DNA fragments were ligated with sequencing adapters. The adapter-containing fragments were treated with sodium bisulfite using the Methylation-Gold Kit (ZYMO Research, CA, United States). The bisulfite-converted DNA fragments were subjected to PCR amplification. The PCR mixture was prepared in a total volume of 50 μL containing NEB Next High-Fidelity 2X PCR Master Mix (25 μL), Index Primer (1 μL), Universal PCR Primer (1 μL), and DNA (23 μL). The PCR cycling conditions were as follows: initial denaturation at 98°C for 30 s, followed by 12 cycles of 98°C for 10 s, 65°C for 75 s, and 72°C for 30 s, with a final extension at 72°C for 5 min. Finally, the purified PCR products were sequenced by Gene Denovo Biotechnology Co. (Guangzhou, China) using the Illumina HiSeq 2,500 platform. Raw sequence data have been submitted to the Sequence Read Archive (SRA) in the National Centre for Biotechnology Information (NCBI) with the bioproject accession number PRJNA905816.

### 2.4. Data filtering and DNA methylation analysis

To generate high-quality clean reads, the raw sequences with >10% unknown nucleotides and or >40% low-quality bases (*q*-value ≤ 20) were eliminated. After filtering, the Bisulfite Sequence Mapping Program (BSMAP) software (version 2.90) was used to map the clean bisulfite reads to the species (GCF_002742125.1) (30). The amount of 5-mC as well as the highest level of cytosine-guanine dinucleotide (CpG) methylation within the whole genome, in each chromosome and in the context of different genomic regions (CG, CHG, and CHH where H represents A, T, or C), were determined using a custom Perl script (31). To evaluate differential methylation patterns across different genomic regions, the methylation activities at 5′-flanking ±2 kb regions of genes or transposable elements were plotted according to the average methylation level of each 100 bp interval. The transposable element insertion and the different types of repetitive regions of the sheep genome were obtained from GCF _ 002742125.1 reference genome is https://ftp.ncbi.nlm.nih.gov/genomes/all/GCF/002/742/125/GCF_002742125.1_Oar_rambouillet_v1.0/. ‘GCF _ 002742125.1 _ Oar _ rambouillet _ v1.0 _ rm.out.gz ‘file.

### 2.5. Differentially methylated region analysis

To assess differential DNA methylation at multiple loci among the study samples, Pearson’s chi-square (*χ*2) test was carried out using methylKit in R (32). The differentially methylated region (DMR) for each sequence context (CG, CHG, or CHH) between two samples was identified based on the following criteria: (i) at least five 5-methylcytosines (5-mCs) in a single sample, (ii) the total depth of sequencing for each CpG site was more than 10X, and the depth of coverage for 5-mCs was more than 4X, (iii) the distance of DMRs(differential methylation regions) ranged from 40 bp to 10 kb, (iv) the distance between two neighboring CpGs was <200 bp, (v) fold-change for the average number of 5-mCs was more than 2, and (vi) false discovery rate was less than 0.05 and *χ*2 value was *p* ≤ 0.05. Putative DMRs overlapping at adjacent 2 kb (upstream or downstream) or body regions of genes or transposable elements were chosen for subsequent analysis.

### 2.6. Functional analysis of differentially methylated genes

To understand the functions of differentially methylated genes (DMGs), gene ontology (GO) and Kyoto Encyclopedia of Genes and Genomes (KEGG) pathway enrichment analyses were conducted. The GO database^1^ was used to identify the core of all significantly enriched gene sets and filter their corresponding biological functions. The KEGG pathway database^2^ was used to determine significantly enriched metabolic pathways and signal transduction pathways. The computed *p*-value was adjusted using the Bonferroni method with a *Q*-value threshold of ≤0.05. GO terms and KEGG pathways that met these requirements were considered as significantly enriched for DMGs.

### 2.7. Interaction network analysis

The interaction network analysis is that Protein–Protein interaction network was identified using String v10[1], which determined genes as nodes and interaction as lines in a network.

[1] Szklarczyk D, Franceschini A, Wyder S, et al. STRING v10: protein–protein interaction networks, integrated over the tree of life[J]. Nucleic acids research, 2014, 43(D1):D447-D452.

## 3. Results

### 3.1. Overview of DNA methylation

DNA methylation is an epigenetic modification that plays a vital role in gene regulation, embryonic development, and tissue differentiation. In this study, whole-genome bisulfite sequencing was used to analyze the genome-wide DNA methylation patterns in muscle of Tan, Hu and Tan-Hu F2 generation sheep. Summary statistics of the whole-genome bisulfite sequencing data analysis were presented in Table 1. Notably, over 751 million (Tan sheep), 594 million (F2 generation) and 721 million (Hu sheep) unique clean reads were generated from WGBS. The mapped ratios were 88.27, 88.24, and 88.74% for Tan sheep, Hu sheep and Tan-Hu F2 generation, respectively, with respective sequencing depths of 35.25, 28.05, and 33.82 million reads. In addition, DMRs with satisfactory depth and high resolution were identified, which covered almost the entire methylome. The ratios of mC, mCHG, and mCHH in Tan sheep and the F2 generation were higher than those in Hu sheep, while the ratio of mCG in the F2 generation was higher than that observed in either pure breed.

**Table 1:** Sequencing information and methylation ratio.

Samples	Tan sheep	Hu sheep	F2 generation
Clean reads	765,031,762	732,060,214	606,863,246
Mapped reads	663,171,015	636,342,020	527,700,617
Percentage of mapped reads (%)	88.27	88.24	88.74
Sequencing depth	35.25	33.82	28.05
mC (%)	3.55	3.18	3.56
mCG (%)	68.84	68.9	70.33
mCHG (%)	0.63	0.43	0.63
mCHH (%)	0.67	0.44	0.67

### 3.2. Characterization of methylated regions

**Figure 1:**
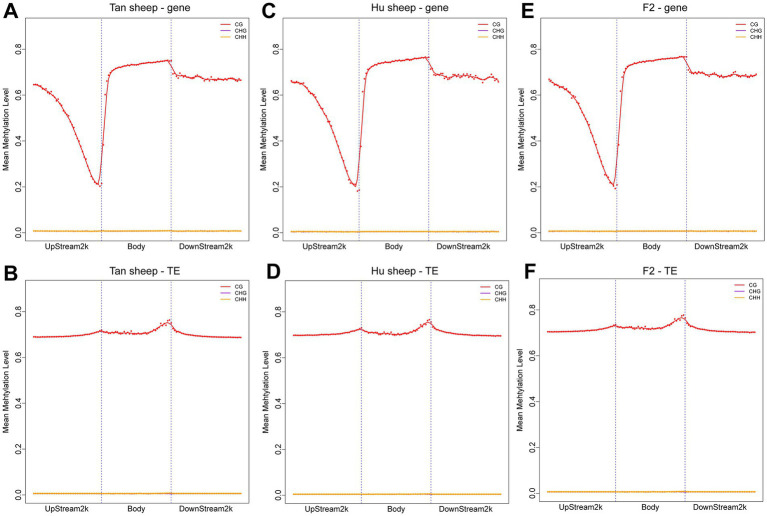
Methylation patterns in the different sheep breeds. DNA methylation levels in the gene body regions and their 2 kb upstream and downstream regions in the genome **(A,C,E)** and DNA methylation levels in the transposable element body regions and their upstream and downstream regions in the genome **(B,D,F)**. Each dot represents the mean methylation level per bin and the corresponding lines indicate the 5-bin moving average. H is defined as A, T, or C. TE: transposable element.

**Figure 2:**
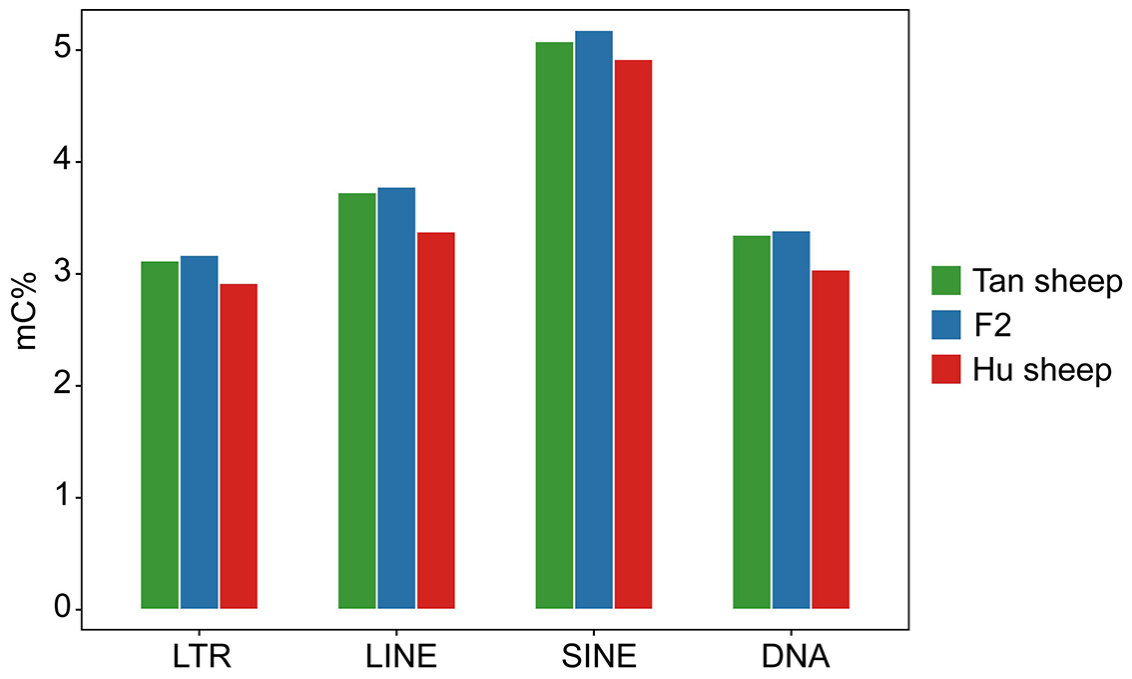
Methylation levels in the different repeat regions. LTR, long-terminal repeat; LINE, long interspersed nuclear element; SINE, short interspersed nuclear element; DNA, DNA repeat.

The distribution of CpG sites across various genomic regions of Tan sheep, Hu sheep and F2 generation sheep is illustrated in Figure 1. Notably, the highest level of DNA methylation was found in the gene body, followed by the downstream region, while the lowest levels in all three types of sheep were observed in the upstream region. In addition, methylation of the repeat regions was also detected across the sheep methylomes. The methylated transposable element or insertion sequence has been shown to be associated with the regulation of gene transcription (33). Further to the observation that the lowest methylation level was detected in Hu sheep, short interspersed nuclear elements showed higher methylation levels in the mC context, across the three sheep breeds (Figure 2).

### 3.3. Identification of differentially methylated regions and differentially methylated genes

**Figure 3:**
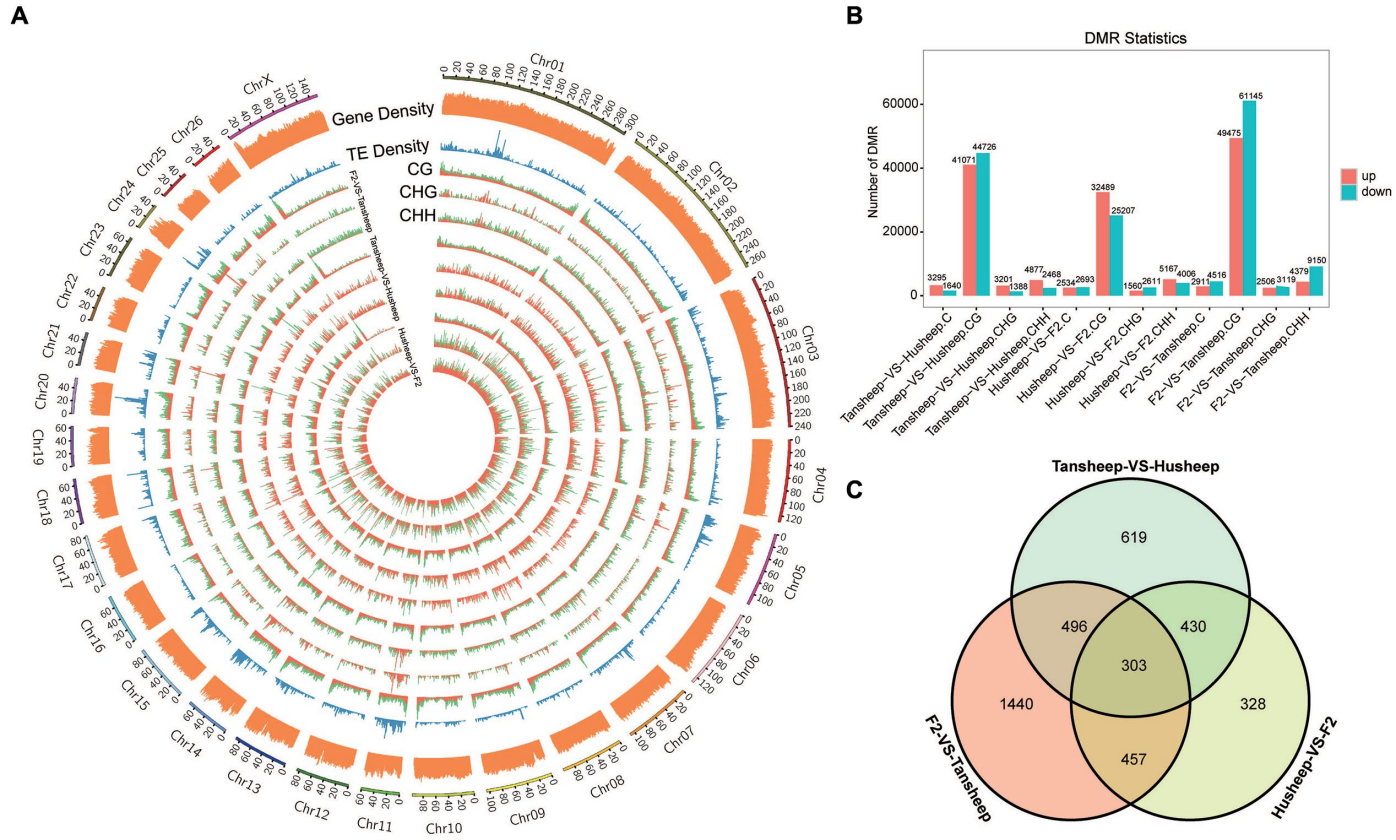
Differences in DNA methylation patterns among Tan sheep, Hu sheep and their offspring. **(A)** Density plot of different 5 mC peaks in each sequence context (CG, CHG, or CHH) among the three comparisons undertaken. H denotes A, T, or C; TE represents transposable element; the outermost rim indicates the chromosome number and scale. **(B)** Total number of DMRs among the three comparisons. **(C)** Unique or shared DMGs among the three comparisons.

To explain the variability observed in longissimus dorsi muscle tissue between Tan and Hu sheep and the resulting Tan-Hu F2 generation, DMRs were identified across their methylomes (Figure 3A). DMRs in each sequence context (CG, CHG, or CHH) were identified based on the following criteria: For CG, CHG, CHH and all C, the numbers of GC in each window were ≥5, ≥5, ≥15, and ≥20, respectively, while the absolute values of the difference in methylation ratios were ≥0.25, ≥0.25, ≥0.15, and ≥0.20 (all *q* ≤ 0.05), respectively. The results showed that more DMRs were identified in the skeletal muscle from the Tan sheep vs. F2 generation compared to Hu sheep vs. F2 generation and Tan vs. Hu sheep. This indicates that the difference in this period of muscle development between Tan sheep and the resulting Tan-Hu F2 generation was larger than that between Hu sheep and the F2 generation (Figure 3B).

Furthermore, the number of overlapping genes between two comparison breeds/F2 was determined according to the following criteria: *p*-value ≤0.05 and |log2(fold-change)| ≥ 2. A total of 4,672, 2,448, and 2,989 methylated genes were identified for Tan sheep vs. F2 generation, Hu sheep vs. F2 generation and Tan vs. Hu sheep, respectively. Venn diagram analysis revealed that 303 differentially methylated genes overlapped among the two breeds and F2 generation (Figure 3C). Meanwhile, 430 genes were methylated in both Tan sheep and F2 generation, but not in Hu sheep.

### 3.4. Go enrichment and KEGG pathway analyses of differentially methylated genes

**Figure 4:**
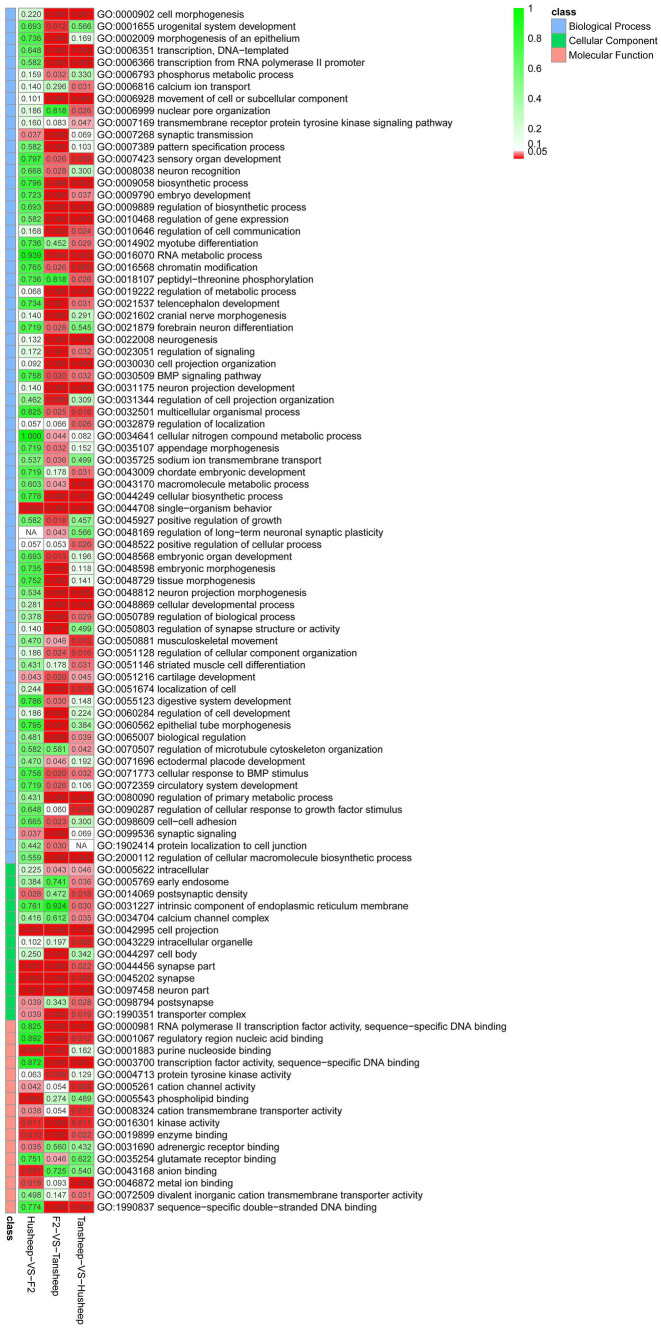
*Q*-values of different GO terms enriched in DMGs.

In this study, the GO enrichment terms associated with DMGs were categorized into 369 functional groups with a *q*-value of ≤0.05 in at least one group (Figure 4). Of the 262 terms enriched for biological process, there were four terms related to muscle development, including myotube differentiation (GO:0014902), myotube cell development (GO:0014904), smooth muscle cell differentiation (GO:0051145), and striated muscle cell differentiation (GO:0051146). Interestingly, there were significant differences in DNA methylation patterns between the Tan and Hu sheep, but no significant differences were found between the original breeds and the F2 offspring. The top-level GO terms annotated genes included forkhead box F1 (*FOXF1*), SWI/SNF-related matrix-associated actin-dependent regulator of chromatin subfamily A member 2 (*SMARCA2*), zinc finger E-box-binding homeobox 1 (*ZEB1*), teashirt zinc finger homeobox 3 (*TSHZ3*), SIX homeobox 4 (*SIX4*), actin alpha 1 (*ACTA1*), myosin heavy chain 11 (*MYH11*), retinoic acid receptor beta (*Rarb*) and retinoid X receptor alpha (*Rxra*).

**Figure 5:**
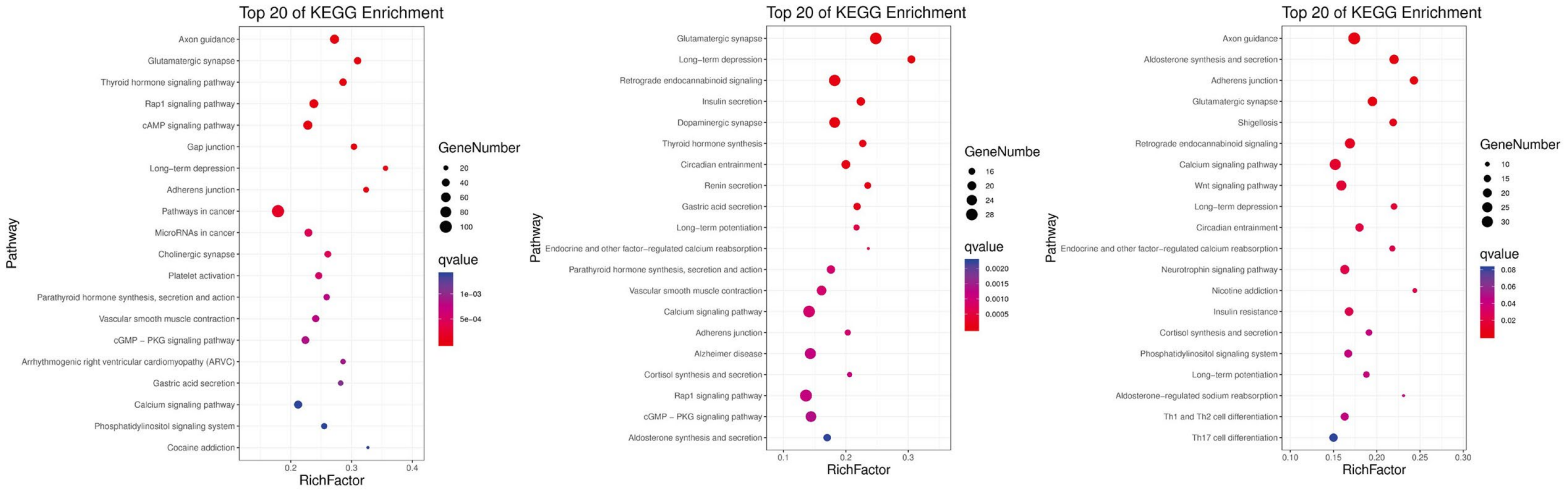
Gene numbers and *q*-values of different KEGG pathways enriched among the three comparisons. Rich factor is the ratio of DMGs in the pathway and the total number of genes in the pathway. The smaller the value of rich factor, the lower the degree of KEGG enrichment.

KEGG pathway analysis was conducted to further reveal the functional roles of DMGs. Overall, the genes were enriched in 129 KEGG pathways that met the criterion of *q*-value ≤0.05 in at least one group (Figure 5). The number of significantly enriched pathways was 82, 54, and 101 in comparisons between Hu vs. F2 generation, Tan vs. Hu and Tan vs. F2 generation, respectively. These results demonstrate that the biological functions regulated by these pathways are crucial for the understanding of differences in meat characteristics between the Tan, Hu and F2 generations and may be involved in muscle development and growth. Regulation of actin cytoskeleton (ko04810) was one of the most relevant pathways related to muscle development. The top-level KEGG pathway annotated genes included myosin light chain kinase (*MYLK*), adenomatous polyposis coli (*APC*), son of sevenless (*SOS*), cholinergic receptor muscarinic 2 (*CHRM2*), guanine nucleotide-binding protein subunit alpha-12 (*GNA12*), fibroblast growth factor (*FGF*), brain-specific angiogenesis inhibitor 1-associated protein 2 (*BAIAP2*), insulin receptor substrate 53 kDa (*IRSP53*) and actinin alpha 1 (*ACTN1*).

### 3.5. Interaction network of differentially methylated genes

**Figure 6:**
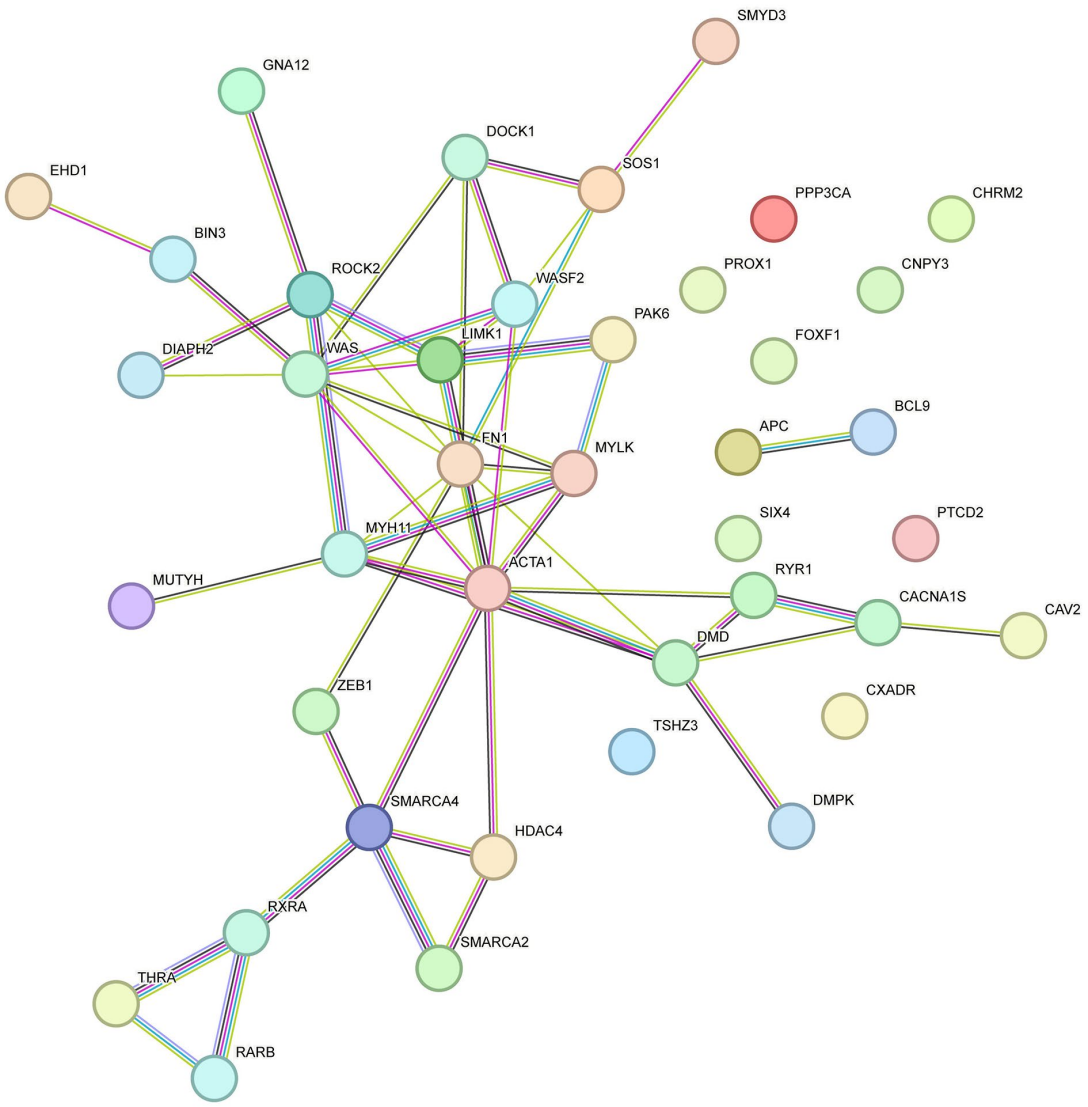
Interactive network of DMGs involved in the developing skeletal muscle.

Previous transcriptome-wide association studies have elucidated the molecular mechanisms of phenotypic variations in sheep. These mechanisms provide a theoretical framework for our study on the association between DMGs and phenotypic differences. STRING-DB v11.0^3^ was employed to construct an interaction network for the genes related to KEGG and GO enrichment. As shown in Figure 6, *ACTA1*, *MYH11*, Wiskott-Aldrich syndrome protein (*WAS*), vav guanine nucleotide exchange factor 1 (*VAV1*), fibronectin 1 (*FN1*) and Rho-associated protein kinase 2 (*ROCK2*) genes were identified as key genes involved in skeletal muscle development.

## 4. Discussion

The skeletal muscle development is closely related to meat yield and quality in animals. The development and growth of muscle involve the proliferation, fusion, and differentiation of myoblast cells into muscle fibers (34). These processes are affected not only by genotype, but also a set of complicated epigenetic regulatory mechanisms, including DNA methylation. In recent years, several studies explored the methylomes of longissimus dorsi, blood and ovarian tissues in different sheep breeds using reduced representation bisulfite sequencing (RRBS) (35), methylated DNA immunoprecipitation sequencing (MeDIP-Seq) (36), Luminometric Methylation Assay (LUMA) (37), and WGBS (38) technologies. Of them, Whole-genome bisulfite sequencing (WGBS) is the most comprehensive DNA methylation sequencing methods, achieving single-base resolution through bisulfite conversion. WGBS have excellent specificity and non-sensitivity, and can obtain almost complete information of methylcytosine (39). For skeletal muscle tissue in sheep, DNA methylation pattern was revealed in Poll Dorset lambs (35), Hu sheep (26), Small Tailed Han and Dorper × Small Tailed Han crossbred sheep (28). Cao et al. (28) identified DMRs between Small Tailed Han and Dorper × Small Tailed Han crossbred sheep and found that these DMRs were enriched in genes associated with metabolic pathways and muscle development. Additionally, they found that certain DMRs were associated with differences in meat quality traits between the two breeds, such as marbling and intramuscular fat content.

In our study, we systematically analyzed the DNA methylation profiles in Tan and Hu sheep and their offspring using WGBS to elucidate the relationship between differential muscle development and DNA methylation. The objective was to identify methylated genes affecting muscle development in Tan sheep. In this study, reads distribution analysis showed that DMRs were highly enriched in the intron regions and repeat elements, which was consistent with previous reports in other animals (40–42). Repeat regions are involved in many biological processes and may be one of the sources of genomic instability, therefore many repeat sequences maintain heterochromatin status through methylation processes. As for the enrichment of intron region or intergenic region, the possible reason is that the proportion of introns is much larger than that of exons, and the probability of methylase binding is greater than exons. A similar methylation pattern was also observed in other animals, such as chicken (43), bovine (44), pig (20). It is most likely that this methylation pattern is conserved among different species.

For all DMGs identified among the three comparisons (Tan sheep vs. F2 generation, Hu sheep vs. F2 generation, Tan sheep vs. Hu sheep), bioinformatics analysis showed significant enrichment in several important pathways related to metabolic and developmental processes. Based on the results of GO enrichment, DMGs were significantly enriched in 369 functional groups, and the functions of these genes were different between the Tan and Hu sheep, which help provide an overview of their phenotypic differences. Network analysis revealed the involvement of differentially methylated genes, namely, ACTA1, MYH11, WAS, VAV1, FN1, and ROCK2, in the developing skeletal muscles. However, these findings were not entirely consistent with those published by Fan et al. (26) showing that adiponectin, C1Q and collagen domain containing (ADIPOQ), cyclin A2 (CCNA2), collagen type I alpha 2 (*COL1A2*), diaphanous related formin 1 (*DIAPH1*), delta-like 1 homolog (*DLK1*), integrin alpha 2 (*ITGA2*), microtubule associated protein tau (*MAPT*), myogenin (*MYOG*), and nuclear receptor 4A1 (*NR4A1*) are the key genes associated with lipid metabolism and muscle development in Hu sheep.

The observed differences in DNA methylation patterns between the three sheep breeds and their F2 generation may reflect variations in the regulation of gene expression during muscle development. DNA methylation can affect gene expression by recruiting proteins that repress transcription or by blocking the binding of transcription factors to DNA. Thus, changes in DNA methylation levels in specific genomic regions, such as gene bodies, can modulate the expression of genes involved in muscle development and differentiation. For example, the identification of DMRs in genes such as *FOXF1, SMARCA2, ZEB1, TSHZ3, SIX4, ACTA1, MYH11*, *Rarb*, and *Rxra* suggests that these genes may be differentially regulated by DNA methylation in the three sheep breeds and their F2 generation. Luo et al. (2022) analyzed genome-wide DNA methylation patterns of muscle and tail-Fat in DairyMeade Sheep and Mongolian Sheep. The results showed that the introns of gene *CAMK2D* (calcium/calmodulin-dependent protein kinase IIδ) demonstrated significant DNA methylation level differences between the two breeds in both muscle and tail-fat tissues, and it may play a crucial role in fat metabolism and meat quality traits (27). The higher levels of DNA methylation in the mC, mCHG, and mCHH contexts in Tan sheep and their F2 generation compared to Hu sheep may be linked to differences in the activity of DNMTs, the enzymes responsible for adding methyl groups to cytosine residues in DNA. Additionally, the observed differences in DNA methylation patterns in repeat regions across the three sheep breeds may reflect variations in the regulation of transposable elements, which are mobile DNA sequences that can disrupt gene function and genome stability. DNA methylation is a key mechanism for silencing transposable elements and preventing their activation. Thus, differences in the DNA methylation levels of transposable elements in muscle tissue may reflect differences in the efficiency of transposable element silencing mechanisms in the three sheep breeds. For example, the higher methylation levels of short interspersed nuclear elements in all three sheep breeds in the mC context may indicate that these transposable elements are actively regulated by DNA methylation during muscle development.

The functions and associations of the other genes highlighted in the present study also indicate some interesting interactions. ACTA1 is a marker gene of myofibroblast formation and is involved in the contraction of smooth muscle. This gene is also associated with smooth muscle cell differentiation and regulated muscle traits in pigs during high temperature-induced fattening (45). ROCK2 is responsible for the differentiation of cardiomyocytes and skeletal myoblasts and is abnormally expressed during muscle growth (46). Caldesmon 1 (CALD1) encodes a bifunctional (calmodulin and actin) binding protein that plays an essential role in the regulation of smooth muscle contraction. MYH11 is a myosin that affects muscle fiber composition and muscle fat content and is therefore a very important gene in meat quality research (47). Our results demonstrated that the methylation level of ROCK2 in Tan sheep was significantly higher than that in Hu sheep. DNA methylation generally had a negative regulatory effect on gene’s expression, therefore the high methylation level of ROCK2 may be one of the important reasons for the lower growth speed of Tan sheep than that of Hu sheep. Meanwhile, other genes have been shown to relate to contraction of smooth muscle and regulation of muscle fat content. Their levels of DNA methylation in mutton of Tan sheep were lower than those of Hu sheep, which might be attributed to the tenderness and good flavor of meat in Tan sheep. The identification of GO terms related to muscle development among the differentially methylated genes suggests that DNA methylation may play a role in regulating the expression of genes involved in myotube differentiation and other aspects of muscle development. The KEGG pathway analysis also revealed enrichment in pathways related to muscle contraction, calcium signaling, and focal adhesion, which are essential for proper muscle function. Therefore, the observed differences in DNA methylation patterns may have functional consequences for muscle development and performance in the different sheep breeds and their F2 generation.

Furthermore, the Tan-Hu F2 population was selected for this study because it provided a suitable platform to identify epigenetic markers associated with the traits of interest. The Tan sheep and Hu sheep are two distinct breeds with different genetic backgrounds, and their F1 offspring would only provide information about the genetic effects of their parents and F1 hybrids could not produce kinds of the phenotypes expected as F2 animals in some growth traits. However, the F2 generation is often used in genetics experiments to study the inheritance of traits. By studying the traits expressed in the F2 generation, we can determine the inheritance patterns of these traits, including whether they are dominant or recessive, and whether they are influenced by multiple genes or environmental factors. In this study, the muscle development of Tan-Hu F2 sheep is significantly accelerated compared to their parents, which may be due to a number of factors, such as differences in gene expression, epigenetic modifications and environmental influences. Overall, this study is the first to provide novel insights into the epigenetic mechanisms of muscle development in Tan sheep, Hu sheep and their F2 offspring. Next, we will further analyze the relationship between these gene methylation and gene expression, and the effect of target gene methylation on expression of genes in downstream pathways in order to further reveal the biological function of methylation.

## 5. Conclusion

In this study, DNA methylation profiles of skeletal muscle in Tan sheep, Hu sheep and their F2 offspring were analyzed. WGBS analysis revealed that a total of 4,672, 2,448, and 2,989 DMGs were identified for Tan sheep vs. F2 generation, Hu sheep vs. F2 generation, and Tan vs. Hu sheep comparisons, respectively. These DMGs were enriched with 262 GO functional groups [myotube differentiation (GO:0014902), myotube cell development (GO:0014904), smooth muscle cell differentiation (GO:0051145) and striated muscle cell differentiation (GO:0051146)] as well as 129 KEGG pathways (*q* ≤ 0.05). The results from the present and previous studies have indicated that the “Regulation of Actin Cytoskeleton” pathway might exert beneficial effects on muscle development in different sheep breeds. Furthermore, the methylation levels of several key genes, such as *WAS, VAV1, ACTA1, MYH11, FN1*, and *ROCK2*, for which the encoded proteins are often responsible for the growth, differentiation, and contraction of skeletal muscles, were significantly distinct between the two breeds. The DNA methylation profiles provided new clues for deciphering epigenetic regulatory mechanisms involved in mutton development and identified novel candidate genes.

## Data availability statement

The datasets presented in this study can be found in online repositories. The names of the repository/repositories and accession number(s) can be found in the article/Supplementary material.

## Ethics statement

The animal study was reviewed and approved by Animal Ethical and Welfare Committee (AEWC) Animal Science Institute/Affiliation of Ningxia Academy of Agriculture and Forestry Science. Written informed consent was obtained from the owners for the participation of their animals in this study.

## Author contributions

CY and JW: writing—original manuscript and data analysis. JZ, YL, YS, AX, and JL: sample collection. JL and AX: data curation. CY and HE: software acquisition. Q-eZ, HE, and CY: experimental design. Q-eZ, HE, and JW: writing—review. YS: visualization. CY, JW, YS, JZ, JL, AX, YL, HE, and Q-eZ have made substantial contributions to the research and the reporting of the results. All authors contributed to the article and approved the submitted version.

## Funding

This research was funded by National Natural Science Foundation of China (No.U21A20246) and the Special Agricultural Project of Tan Sheep Breeding in Ningxia (Molecular Marker Assisted Breeding of Tan Sheep, no. 2013NYYZ0404).

## Conflict of interest

The authors declare that the research was conducted in the absence of any commercial or financial relationships that could be construed as a potential conflict of interest.

## Publisher’s note

All claims expressed in this article are solely those of the authors and do not necessarily represent those of their affiliated organizations, or those of the publisher, the editors and the reviewers. Any product that may be evaluated in this article, or claim that may be made by its manufacturer, is not guaranteed or endorsed by the publisher.

## Footnotes

^1^ http://www.geneontology.org/

^2^ http://www.kegg.jp/kegg/

^3^ https://string-db.org/
